# The relevance of using 3D cell cultures, in addition to 2D monolayer cultures, when evaluating breast cancer drug sensitivity and resistance

**DOI:** 10.18632/oncotarget.9935

**Published:** 2016-06-10

**Authors:** Susan Breslin, Lorraine O'Driscoll

**Affiliations:** ^1^ School of Pharmacy and Pharmaceutical Sciences & Trinity Biomedical Sciences Institute, Trinity College Dublin, Dublin 2, Ireland

**Keywords:** 3D cell culture, monolayer culture, breast cancer, targeted drugs, drug resistance mechanisms

## Abstract

Solid tumours naturally grow in 3D wherein the spatial arrangement of cells affects how they interact with each other. This suggests that 3D cell culture may mimic the natural *in vivo* setting better than traditional monolayer (2D) cell culture, where cells are grown attached to plastic. Here, using HER2-positive breast cancer cell lines as models (BT474, HCC1954, EFM192A), the effects of culturing cells in 3D using the poly-HEMA method compared to 2D cultures were assessed in terms of cellular viability, response/resistance to anti-cancer drugs, protein expression and enzyme activity. Scanning electron microscopy showed the morphology of cells in 3D to be substantially different to those cultured in 2D. Cell viability in 3D cells was substantially lower than that of cells in 2D cultures, while 3D cultures were more resistant to the effects of HER-targeted (neratinib) and classical chemotherapy (docetaxel) drugs. Expression of proteins involved in cell survival, transporters associated with drug resistance and drug targets were increased in 3D cultures. Finally, activity of drug metabolising enzyme CYP3A4 was substantially increased in 3D compared to 2D cultures. Together this data indicates that the biological information represented by 3D and 2D cell cultures is substantially different i.e. 3D cell cultures demonstrate higher innate resistance to anti-cancer drugs compared to 2D cultures, which may be facilitated by the altered receptor proteins, drug transporters and metabolising enzyme activity. This highlights the importance of considering 3D in addition to 2D culture methods in pre-clinical studies of both newer targeted and more traditional anti-cancer drugs.

## INTRODUCTION

In the drug development process and, indeed, any laboratory setting wishing to mimic as best as possible the *in vivo* environment in their pre-clinical studies, it is important that the experimental model of the disease being used in testing is as true to life as possible. For breast cancer research, it is important that the cell models used in research to further our knowledge of the disease represent the disease in terms of expression of target receptors, drug transporters and proteins essential for cell survival and growth, as well as activity of enzymes responsible for drug metabolism.

The natural manner in which solid tumours grow *in vivo* is three-dimensional. This suggests that growing cancer cells in 3D mimics the *in vivo* environment better than traditional 2D cell culture due to the ability of the cells to form cell-cell interactions and develop into 3D structures, as opposed to growing flat and attached to cell culture-grade plastic. This suggests that 3D culture is more *in vivo*-like than 2D cultures. This theory is supported by the fact that cells grown as 2D cultures lose some of their natural functional abilities; however these changes can be restored simply by growing cells in 3D again. For example, immortalised human hepatocyte HepG2 cells are routinely used as a liver model for drug toxicity testing *in vitro.* However, when these cells are grown in traditional 2D culture they lose substantial amounts of CYP450 enzyme mRNA and activity, that are critical to liver cells' ability to metabolise drugs [1, 2], thus limiting their ability to effectively mimic liver function and predict drug toxicity in humans. Ramaiahgari *et al*. [3] however, found that growing the HepG2 cells in 3D using an ECM hydrogel resulted in restored phenotypic characteristics of hepatocytes as they occur *in vivo* in terms of proliferation, formation of bile canaliculi, and increased levels of CYP3A4 mRNA and activity; which are, ultimately, the liver-like properties of the cells. Together this data suggests that 3D cell culture is more similar and relevant to the *in vivo* setting than 2D cell culture.

How cells are typically grown in 2D and how they can be grown in 3D, in the context of their natural environment, has been reviewed by us [4] and so is not detailed again here. The field of 3D cell culture research is, however, still in its infancy in comparison to the knowledge established on 2D cell culture. Further research is essential to further characterise this method of growing cells *in vitro* for evaluating anti-cancer drugs. Thus, the aim of this study was to culture cells under conventional 2D conditions and also using the forced-floating poly-HEMA method of 3D culture in order to characterise differences observed between the two methods. More specifically, using three HER2-overexpressing breast cancer cell lines (BT474, HCC1954 and EFM192A) we aimed to investigate differences in expression of cell survival proteins, drug targets and drug transporters between 2D and 3D cells. Additionally, cell viability, response to drug treatment and CYP3A4 activity were compared in both cell culture methods.

## RESULTS

### Different morphology of cells grown in 2D versus 3D

**Figure 1:**
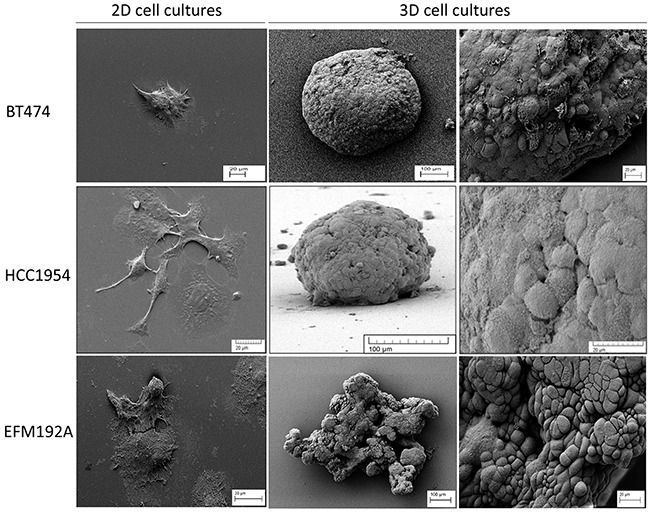
Different morphology of each cell line in 2D and 3D culture SEM images show how the morphology of cells differs substantially when grown in 2D compared to 3D cultured cells. For all lines, cells grew attached to the cover-slip when using standard culture methods. Both BT474 and HCC1954 cells form a tightly packed spheroid when grown in 3D, while EFM192A cells form a less organised 3D structure when cultured under the same conditions. Scale bars are shown on all images.

SEM imaging revealed an in depth view of the difference in morphology of cells grown with the 2D and 3D culture methods (Figure 1). BT474 cells grow together in patches in 2D culture but, when grown under forced-floating conditions, they form uniform spheroids with a relatively smooth surface. HCC1954 cells in 2D exist more independently, as opposed to in groups/colonies, and are more spread out than BT474 cells. HCC1954 3D cultures form tight spheroids, but with a less smooth surface than BT474 3D cells. BT474 and HCC1954 cells, when grown in 3D, appear to secrete an extracellular matrix (ECM) [5] which smoothens the surface of the sphere and makes it difficult to distinguish individual cells. Pores appear to form in the surface of these spheroids. EFM192A cells grow similarly to BT474 cells in 2D in that they grow in patches; however, these cells have a more rounded shape. EFM192A cells cultured under forced-floating conditions form a 3D structure; however, their 3D shape is not as tight or homogenous as either the BT474 or HCC1954 spheroids.

### Altered cell viability in 2D and 3D cell culture

**Figure 2:**
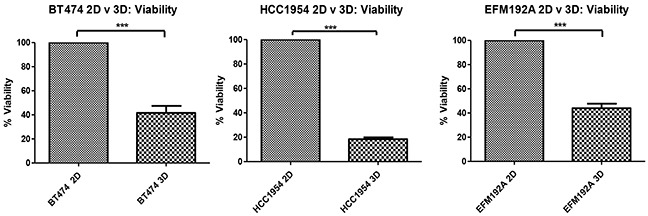
2D compared to 3D cell viability Starting with the same cell numbers and time of culture, cells cultured in 3D have significantly decreased viability compared to those cultured as 2D monolayers. Graphs represent triplicate biological repeats and are displayed as mean ± SEM, where ***p < 0.001.

After 6 days of culturing cells that had been seeded at the same density (specific for each cell line) in 2D, cellular levels of ATP were measured as an indication of cell viability (Figure 2). Results showed that BT474 3D cell viability was only 41.6±5.9% of that measured in BT474 2D cells (p=5.8×10^−4^). Similarly, HCC1954 3D cell viability was only 18.4±1.5% of that measured in HCC1954 2D cells (p=6.3×10^−7^) and EFM192A 3D cell viability was 44±3.7% that of the EFM192A 2D cells (p=1.1×10^−4^).

### Decreased drug efficacy in 3D compared to 2D cultures

**Figure 3:**
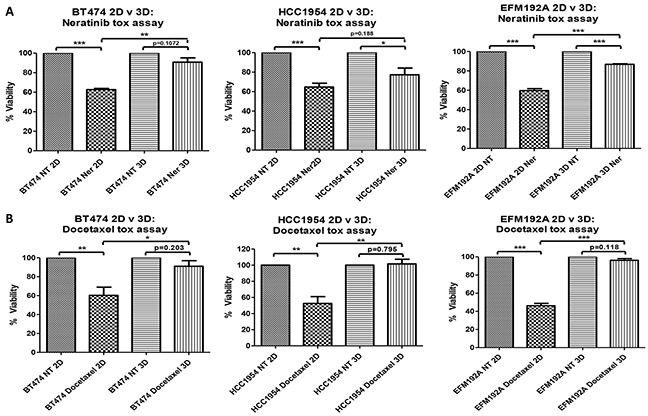
2D v 3D cell sensitivity to neratinib and docetaxel 3D cells are less sensitive to the effects of **A.** neratinib (Ner) and **B.** docetaxel compared to 2D cultured cells. Graphs represent triplicate biological repeats and are displayed as mean ± SEM, where *p < 0.05; **p < 0.01; ***p < 0.001. NT = not treated with drug.

Efficacy of the HER-targeted drug, neratinib, was compared between cells cultured in 2D and 3D (Figure 3(A)). For BT474, 2D cultured cells treated with neratinib had 62.7±1.2% cell survival compared with untreated with drug (NT) cells (p=7.3×10^−6^), while BT474 3D cells treated with the same concentration of neratinib showed 90.8±4.5% cell survival compared to untreated 3D cells (p=0.107), indicating very limited sensitivity to neratinib in the 3D cultures. The significantly (p=0.004) increased difference in cell survival, in the presence of a fixed concentration of neratinib, between 3D and 2D cells was 28.1±5.4%. A similar trend was seen in both HCC1954 and EFM192A cells where 2D cells treated with fixed concentrations of neratinib showed 64.7±3.9% (p=8.0×10^−4^) and 59.7±2.1% (p=4.2×10^−5^) cell survival, respectively, compared to their untreated 2D counterparts, while neratinib treated 3D cells maintained a higher 77.3±6.9% (p=0.031) and 86.8±0.6% (p=2.3×10^−5^) survival, respectively. In these cell lines the difference in 3D and 2D cell survival following neratinib treatment was 12.6±5.3% (p=0.188) and 27.1±2.7% (p=2.3×10^−4^).

The efficacy of a classical chemotherapeutic drug, docetaxel, was also reduced in 3D compared to 2D cultures (Figure 3(B)). BT474 cells cultured in 2D and treated with docetaxel had 60.3±8.7% survival compared with untreated cells (p=0.01), while 3D cells treated with the same concentration of docetaxel maintained 91±5.9% cell survival compared to untreated 3D cells (p=0.203). The significant (p=0.043) difference in cell survival between 3D and 2D cells following the same docetaxel treatment was 30.7±2.8%. A similar trend was seen in both HCC1954 and EFM192A cells where 2D cells treated with fixed concentrations of docetaxel showed 52.3±8.5% (p=0.005) and 46.2±2.6% (p=4.2×10^−5^) cell survival, respectively, compared to untreated 2D cells, while 3D cells treated with the same concentration of docetaxel maintained a higher rate of survival at 101.6±5.7% (p=0.795) and 96.2±1.9% (p=0.118), respectively. For these cell lines, the increase in 3D cell survival compared to 2D cell survival in response to docetaxel treatment was 49±3.1% (p=0.009) and 50±2.5% (p=1.0×10^−4^).

### Cell survival pathways in 2D and 3D cell culture

**Figure 4:**
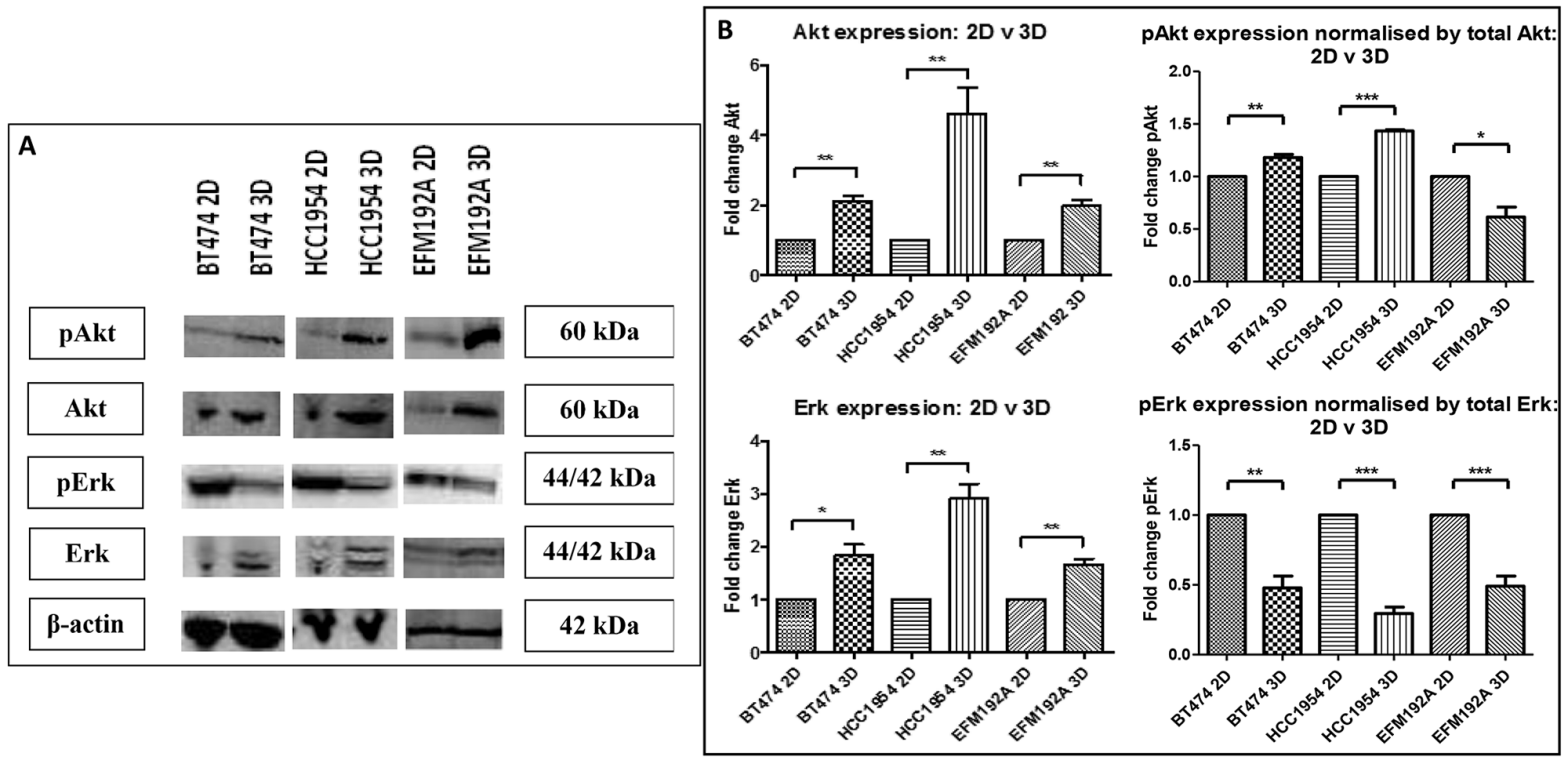
Immunoblots of pAkt, Akt, pErk and Erk expression in 2D and 3D cultures **A.** Akt, pAkt and Erk level were all significantly higher in 3D cultures of each of the cell lines compared to their 2D counterparts, while pErk levels were significantly reduced. **B.** Densitometry of respective immunoblots. Graphs represent triplicate biological repeats and are displayed as mean ± SEM, where *p < 0.05; **p < 0.01.

The effects of different methods of cell culture on Akt and Erk, important components of cell survival pathways, were evaluated using immunoblots (Figure 4(A)). Akt expression was found to be increased in cells cultured in 3D compared to those in 2D, where BT474 3D, HCC1954 3D and EFM192A 3D cells had 2.1±0.2 fold (p=0.002), 4.6±0.8 fold (p=0.009) and 2.0±0.2 fold (p=0.004), respectively, more Akt than their 2D counterparts. In keeping with this, pAkt was over-expressed when normalised to total Akt in 3D cells where BT474 3D and HCC1954 3D cells had 1.2±0.03 fold (p=0.003) and 1.4±0.01 fold (p=2.1×10^−6^), respectively, more pAkt than their corresponding 2D cells. pAkt in EFM192A 3D versus 2D cultured cells decreased to 0.6±0.1 (p=0.015). Similarly, 3D cells had higher levels of Erk expression where BT474 3D, HCC1954 3D and EFM192A 3D cells had 1.8±0.2 fold (p=0.013), 2.9±0.3 fold (p=0.002) and 1.7±0.1 fold (p=0.005), respectively, more Erk than their 2D counterparts. Conversely, levels of pErk decreased in 3D cells in comparison to 2D cultures when normalised to total Erk in BT474, HCC1954 and EFM192A cell lines to 0.48±0.1 (p=0.004), 0.29±0.05 (p=1.1×10^−4^) and 0.49±0.08 (p=0.002). β-actin, established as unchanged in each of the three cell lines regardless of culturing conditions, was used as a loading control for immunoblots. The corresponding densitometry graphs for these immunoblots are displayed in Figure 4(B).

### HER-family drug target expression in 2D and 3D cell culture

**Figure 5:**
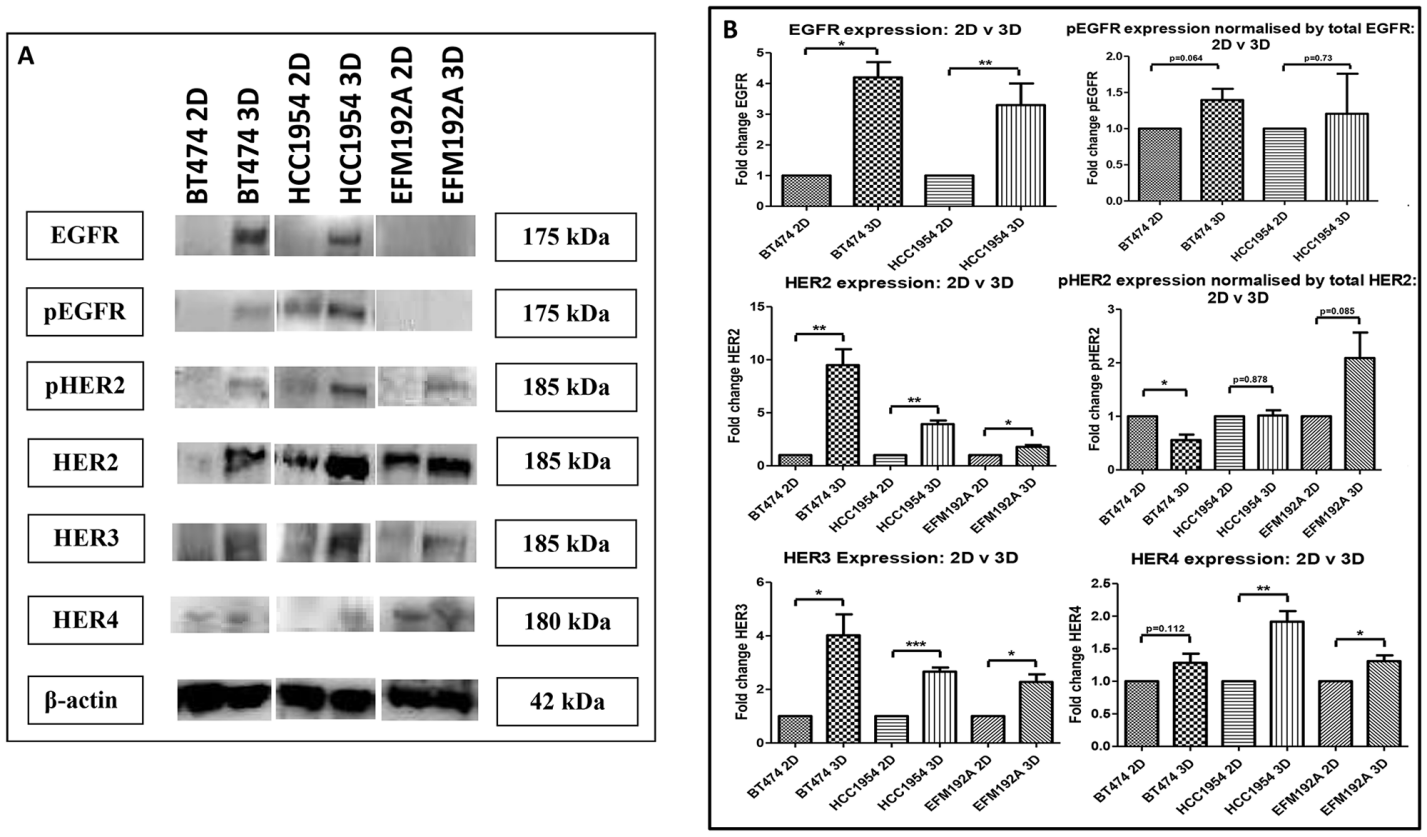
Immunoblots of EGFR family members in 2D and 3D cultures **A.** EGFR, pEGFR, HER2, pHER2, HER3 and HER4 were all increased in 3D cultured cells compared to their 2D counterparts, with the exception that EGFR/pEGFR were undetected in EFM192A cells grown in either format. **B.** Densitometry of respective immunoblots. Graphs represent triplicate biological repeats and are displayed as mean ± SEM, where *p < 0.05; **p < 0.01; **p < 0.001.

The effects of culturing cells in 3D compared to 2D on the expression of the EGFR family of receptors (targets of HER-targeted therapies), was investigated using immunoblots (Figure 5(A)). EGFR expression was found to be increased in both BT474 3D and HCC1954 3D cells by 4.2±0.5 fold (p=0.003) and 3.3±0.8 fold (p=0.036), respectively, compared to their 2D counterparts. Similarly, pEGFR expression was increased (but not significantly) when normalised to total EGFR in both BT474 3D and HCC1954 3D cells by 1.4±0.15 fold (p=0.063) and 1.2±0.55 fold (p=0.73), respectively, compared to their 2D control cells. EGFR and subsequently pEGFR were not found to be expressed by EFM192A cells. Of note, the analysis for EFM192A cells was performed on the same blots as that of the BT474 and HCC1954 cells, which showed that the EGFR and pEGFR antibodies/conditions were suitable and so supports this observation with EFM192A 2D and 3D cells.

HER2 expression was found to be increased in BT474 3D, HCC1954 3D and EFM192A 3D cells by 9.5±1.5 fold (p=0.005), 3.9±0.3 fold (p=0.001) and 1.8±0.2 fold (p=0.013), respectively, compared to their 2D cells. pHER2 expression was assessed by normalising to total HER2. pHER2 was reduced to 0.56±0.1 (p=0.012) in BT474 3D compared to 2D cultures. While there was no change in pHER2 expression in HCC1954 3D compared to 2D cells, 1.02±0.1 (p=0.878). pHER2 was increased in EFM192A 3D cells by 2.1±0.48 fold (p=0.084) in comparison to cells grown in 2D.

HER3 expression was increased in BT474 3D, HCC1954 3D and EFM192A 3D cells by 4.0±0.8 fold (p=0.018), 2.7±0.15 fold (p=2.9×10^−4^) and 2.3±0.3 fold (p=0.011), respectively, in comparison to their 2D counterparts. HER4 expression was also increased (*albeit* only slightly) in 3D cell cultures compared to 2D, where BT474 3D, HCC1954 3D and EFM192A 3D HER4 expression increased by 1.28±0.1 (p=0.112), 1.91±0.2 (p=0.005) and 1.31±0.1 (p=0.025). See Figure 5(B) for the corresponding densitometry for these immunoblot.

### Drug efflux pump expression in cells grown using 2D and 3D culture methods

**Figure 6:**
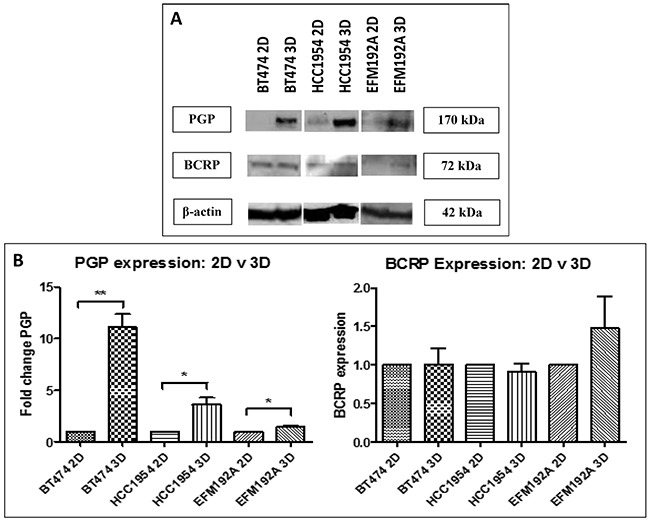
Immunoblots of drug transporter expression in 3D compared to 2D cultures **A.** P-glycoprotein (PGP) level were increased in 3D cultures of all three cell lines, but breast cancer resistance protein (BCRP) levels were not consistently changed between 3D and 2D. **B.** Densitometry of respective immunoblots. Graphs represent triplicate biological repeats and are displayed as mean ± SEM, where *p < 0.05; **p < 0.01.

Expression of two drug transporters, multiple-drug resistance p-glycoprotein (PGP) and breast cancer resistance protein (BCRP) was compared between cells cultured in 2D and 3D (Figure 6(A)). PGP expression was found to be increased by 11.1±1.3 fold (p=0.001), 3.6±0.7 fold (p=0.02) and 1.4±0.15 fold (p=0.049), in BT474 3D, HCC1954 3D and EFM192A 3D cells, respectively, compared to their relative expression in 2D cultures. BCRP was not found to be significantly altered in 3D compared to 2D cells. BT474 3D cells had 1.01±0.2 (p=0.962) fold more BCRP compared to BT474 2D cells, HCC1954 3D cells were found to have 0.9±0.1 less (p=0.457) BCRP compared to HCC1954 2D cells, while EFM192A 3D cells had 1.5±0.5 fold (p=0.309) more BCRP than their corresponding 2D cells. The corresponding densitometry graphs for these immunoblots are displayed in Figure 6(B).

### CYP3A4 activity and protein expression in 2D and 3D cultured cells

**Figure 7:**
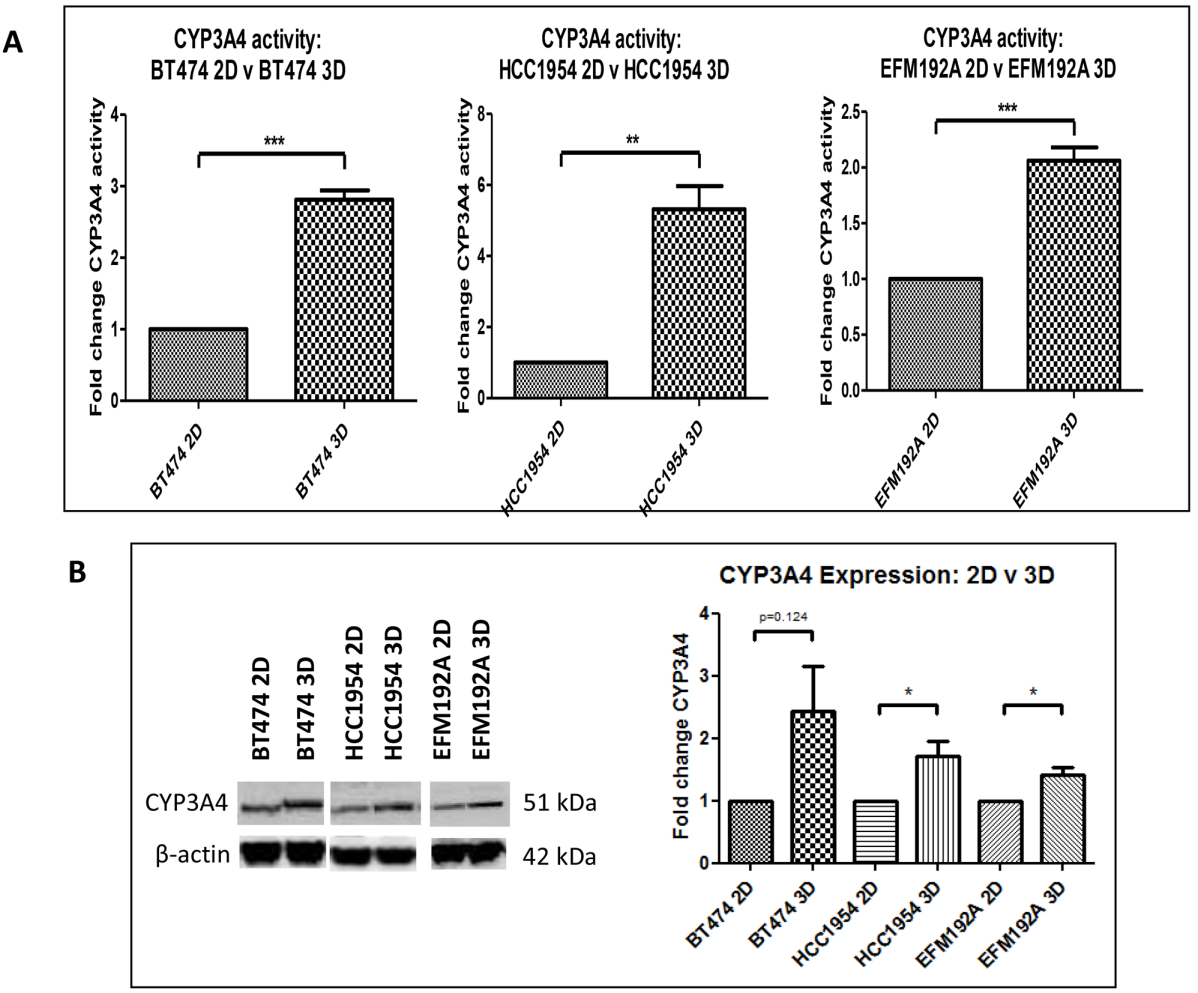
CYP3A4 activity and protein in 3D compared to 2D cells **A.** Cells grown in 3D have significantly higher CYP3A4 activity compared to 2D cultured cells. **B.** CYP3A4 protein quantities are increased in 3D cells compared to cells grown in 2D. Graphs represent triplicate biological repeats and are displayed as mean ± SEM, where *p<0.05, **p < 0.01***p < 0.001.

CYP3A4 activity in 2D and 3D cells was normalised to cell viability, in order to investigate if culturing cells using different methods affects the activity of this drug metabolising enzyme (Figure 7). BT474 3D cells were found to have 2.8±0.1 fold more CYP3A4 activity compared to that detected in BT474 2D cells (p=1.5×10^−4^). Similarly, HCC1954 3D and EFM192A 3D cells had 5.3±0.7 fold (p=0.003) and 2.1±0.1 fold (p=8.3×10^−4^) more CYP3A4 activity, respectively, than their corresponding 2D cells. Additionally, CYP3A4 protein levels were evaluated and it was found that 3D cells expressed more CYP3A4 than cells grown as 2D cultures. Specifically, BT474 3D, HCC1954 3D and EFM192A 3D cells had 2.4±0.7 (p=0.124), 1.7±0.2 (p=0.04) and 1.4±0.1 (p=0.02) more CYP3A4 than their corresponding 2D cells.

## DISCUSSION

The poly-HEMA method of 3D cell culture prevents cell attachment to culture plastic. This encourages the cells to adhere to each other instead and thus form 3D cell structures. This is a relatively simple (*albeit* more labour-intensive than traditional 2D/monolayer culture) and cheap method of 3D cell culture that allows for the generation of a single spheroid per well of a 96 well plate and could be integrated into most laboratory settings.

The morphology of cells grown using this technique is substantially different in comparison to traditional monolayer methods. In 2D culture, cells grow flat and spread out on the cell culture plastic, whereas in the 3D cultures cells aggregate together and form spheroids which have also been reported by others using ECM [6, 7] and poly-HEMA [8] methods for HCC1954, BT474 and EFM192A cells, respectively. This ability to form 3D structures is not a characteristic of all cell lines, as MDA-MB-231, MDA-MB-468, SKBR3 and MDA-MB-361 cells have been shown to form only loosely bound, flat aggregates when grown using the poly-HEMA method [9]. Furthermore, our SEM images of HCC1954 and BT474 3D cells indicated that these 3D spheroids form what is apparently an ECM on their surface through which pores exist. This characteristic of pores in the surface of spheroids has been observed with HepG2 liver cells cultured in 3D using the hanging-drop method [5]. Interestingly, E-cadherin, a cell adhesion molecule, does not appear to play an essential role in the maintenance of the 3D spheroids structure (see Supplementary Figure S1).

The reduced cell viability observed in 3D cell versus 2D cell cultures may be partly contributed to simply by space (of course, as outlined later, reduced pErk levels in the 3D cells may also be a contributing factor). Monolayer/2D cells typically grow continuously, provided they have the space to do so, but 3D cultures do not follow the same growth pattern. Due to the logistics of cells growing in a 3D formation, the cells at the centre do not receive oxygen (*i.e.* experience hypoxia) or nutrients to the same extent as outer cells and so may die as a consequence of this, resulting in the necrotic core [10]. We found that apoptotic markers Caspase 3, Caspase 7 and Caspase 9 were increased in 3D cells compared to 2D cells, which supports this theory that cells are dying and thus contributing to a hollow core (see Supplementary Figure S2). While the outermost layer of cells in a spheroid can proliferate, the middle layer of cells are typically reported as being comprised of quiescent cells [10]. As a result, although the same number of cells can be seeded in 2D and 3D, each of the 2D cells is likely to continue to divide as are their progeny (while space allows), while only the cells that form the outer layer of 3D cell cultures are able to proliferate. It is in this way that the viability of our 3D cells is likely to be substantially reduced compared to 2D cultured cells. The reduced viability in 3D, compared to 2D, cells has also been reported by other groups, where MCF7 breast cancer cells grown in 3D using collagen gels had substantially reduced proliferation compared to cells grown in monolayer [11]. Similarly, the proliferation of a range of colon cancer cell lines (Caco-2, DLD-1, HT-29, SW-480, LoVo, COLO-205 and COLO-206f) grown in 3D using Matrigel was decreased compared to growth observed in 2D cells [12].

*In vitro* drug testing using 2D cell culture is typically used to establish which, out of a range of lead compounds, are most effective. Consequently, we investigated the efficacy of representative targeted (neratinib) and classical (docetaxel) anti-cancer drugs in both 2D and 3D cultures in order to determine any differences that culture methods may have on drug efficacy *in vitro*. We established that cells grown in 3D show a degree of resistance to the effects of both drugs, in comparison to 2D cultures. We also found that a number of proteins involved in cell growth and survival (Akt, pAkt, Erk and EGFR family of receptors), those that are common drug targets (EGFR and HER2) and a well-established drug-transporter that confers multiple-drug resistance (PGP), are all increased in cells grown in 3D. It is likely that the increases in these proteins contribute to the increase in innate resistance to the drugs investigated here, as the overexpression of these proteins has previously been associated with facilitating acquired drug-resistance. While EMT has previously been shown to be involved in drug resistance [13], our investigation of EMT markers, ALDH and β-catenin, showed no link between 3D drug resistance and EMT (see Supplementary Figure S1).

Decrease in drug efficacy in 3D cells observed here has also been reported by other groups. Specifically, Wen *et al.* [14] reported that 3D cultures (in Matrigel) of MIAPaCa-2 and PANC-1 pancreatic cancer cell lines, when treated with gemcitabine and 5-FU, had a higher resistance to the effects of these drugs in comparison to cells grown in 2D. Similarly, Ponce de León *et al*. [15] found that lung cancer INER-51 cells grown in 3D using agarose, compared to 2D cells, had an increase in resistance to the effects of doxorubicin, etoposide and methotrexate. Lovitt *et al*. [16] reported that the efficacy of epirubicin, vinorelbine and paclitaxel was reduced in breast cancer cells grown in 3D using Matrigel when compared to cells grown in monolayers. Additionally, de le Puenta *et al*. [17] also demonstrated that multiple myeloma cells grown in 3D (using Matrigel and AlgiMatrix), compared to their 2D cultures, are less sensitive to bortezomib and carfilzomib. Together, this data indicates that cells cultured using 3D methods have a higher innate resistance to anti-cancer agents in comparison to cells grown in 2D. Advancing on this and including HER-targeted drug, neratinib, as well as traditional chemotherapy, support this increased innate resistance being relevant also in breast cancer and to different classes of anti-cancer drugs.

While we found that expression of Akt, pAkt and Erk were all increased and pErk was down-regulated in all 3D compared to 2D cell cultures, other groups have also seen that expression of these proteins can change with altered growth methods. Konishi *et al*. [18] found that ovarian cancer ES-2 cells, grown in 3D using the forced-floating technique, had increased levels of Akt and pAkt, and while levels of both Erk and pErk decreased with 3D cell culture. Caco-2 cells, cultured as 3D spheroids had increased Erk and pErk expression in 3D compared to 2D cultures, while Akt was increased but pAkt was decreased in 3D [12]. In our study perhaps the increased levels of Akt and pAkt observed in our 3D cells is, at least partially, responsible for increased resistance to anti-cancer drugs. It may be that the decrease in viability in cells cultured in 3D cell versus 2D is due to their decreased levels of pErk. Collectively, however, the substantial differences in expression of these proteins by cells grown in 3D and 2D indicates that these methods of culture do not produce the same basic biological information within cells.

Similarly, expression of the EGFR family was found to differ between 2D and 3D cells. Specifically, EGFR, pEGFR, HER2, HER3 and HER4 were all found to be detected at higher levels in 3D cells in comparison to 2D cells, while results for pHER2 were variable. As an increase in EGFR and HER2 has previously been associated with resistance to lapatinib [19, 20], it is perhaps the case that the overexpression of these receptors in 3D cells mediates part of the resistance of the cells cultured in 3D to the effects of neratinib and docetaxel. Other groups, studying different cell line models to those included here, have also observed differential expression of these receptors between 2D and 3D cells. For example, Konishi *et al*. [18] also found that EGFR and pEGFR increase in ovarian ES-2 cells grown in 3D compared to 2D cells. Fong *et al.* [21] found HER2 was increased in Ewing sarcoma, EWS, cells grown in 3D using scaffolds compared to 2D cells. They also measured HER2 expression in *in vivo* Ewing sarcoma TC-71 xenographs and found that this increased expression of HER2 in 3D cells is more representative of HER2 levels seen in the *in vivo* xenographs than the lower HER2 expression levels detected in 2D cells. While Pickl and Reis [22] did not find any differences in HER2 expression between SKBR3 2D and 3D cells, they reported that pHER2 levels in 3D cultures to be increased. Weigelt *et al*. [23] demonstrated that HER3 expression differs between 2D and 3D cultures. Specifically, they found that HER3 expression is increased in HCC1569 breast cancer cells grown in 3D using Matrigel compared to 2D cells; however, they also showed that HER3 expression is decreased in AU565, SKBR3 and BT549 cells grown in 3D compared to 2D cultures. Hua *et al*. [24] found that NBL neuroblastoma cells grown in 3D using the poly-HEMA method have higher levels of HER4 compared to cells grown in 2D.

The expression of PGP, but not BCRP, was found to be increased in 3D cells in comparison to 2D cells. Increased levels of this drug transporter could reduce the levels of substrate drugs within cells in 3D, therefore this increased PGP expression may contribute to the decrease in drug efficacy observed in 3D cultures. Doublier *et al.* [25] had similar observations and found that growing MCF7 cells in 3D resulted in higher PGP levels compared to cells grown in 2D. They also found that this increase PGP levels in 3D cells corresponded to a decrease in doxorubicin accumulation within the cells, in comparison to cell cultured in 2D.

CYP3A4 is a cytochrome p450 enzyme that plays a role in the metabolism of at least 50% of drugs being used in the clinical setting [26]. Also, it has been shown that elevated levels of this enzyme may contribute to drug resistance [27]. Due to the prominence of CYP3A4 in drug metabolism and drug resistance, the activity of this enzyme was measured and it was found that 3D cells have higher CYP3A4 activity than 2D cells. This result was supported by immunoblots that showed that 3D cells had higher quantities of CYP3A4 protein than cells grown in 2D. Neratinib [28] and docetaxel [29] are both substrates for CYP3A4 and, as these drugs exhibit decreased efficacy in 3D cells, it is likely that the increased CYP3A4 activity in 3D cells is at least partially responsible for the decreased drug efficacy observed in 3D cells. The increased enzyme activity in 3D cultures observed here has also been reported for liver cells, where HepG2 cells cultured in 3D using Matrigel had higher CYP3A4 activity compared to that of 2D cultures [3]. Additionally, Takahashi *et al*. [30] found that CYP3A4 mRNA levels were increased in HepG2 cells grown in 3D using the hanging drop method, in comparison to 2D cells. However, to the best of our knowledge, this is the first report of this observation in breast cancer. Future studies blocking CYP3A4 activity and assessing the knock-on effects on drug sensitivity/resistance are now warranted. Furthermore, further advancement of these studies to testing resistance of the spheroids transferred in to extracellular matrix, inducing cell invasion, could be interesting.

In conclusion, *in vitro* cell culture is an essential method for biological sciences and the drug development process. So, the methods used must be of the highest quality and relevance to the *in vivo* situation of the biological processes being investigated. The data presented here indicates that the biological information represented by 2D and 3D cell cultures is fundamentally different. We showed that the expression of a number of proteins involved in cell survival, drug targeting and drug transporters and the protein expression and activity of an enzyme that plays a major role in drug metabolism are all increased in 3D compared to 2D cultures. Additionally, we found that drug efficacy is reduced in cells grown in 3D that show a higher innate resistance to both targeted and classical chemotherapeutic drugs, compared to 2D cultures. This increase in resistance is likely facilitated by the increased protein expression and drug metabolising enzyme activity observed. The fact that, with very few exceptions, the observations made were consistent across all 3 cell line models used gives a level of confidence that these observations are real, not cell line specific, and high-lights the importance of considering 3D, not only 2D, cultures in pre-clinical studies of anti-cancer drugs.

## MATERIALS AND METHODS

### 2D and 3D cell culture

BT474 and HCC1954 cells were obtained from ATCC, while EFM192A cells were obtained from the Leibniz-Institut DSMZ. Cells were cultured in RPMI supplemented with 1% L-glutamine and 10% FBS. Cells described as 2D cells refer to those grown in the typical cell culture manner, attached to T25cm^2^ or T75cm^2^ (Corning) plastic cell culture flasks. Cells referred to as 3D cells were grown under ‘forced floating’ 3D cell culture conditions [4], where round-bottomed 96 well plates (Corning) were coated with poly-HEMA (Sigma-Aldrich). Poly-HEMA was prepared by dissolving 1.2mg poly-HEMA in 100ml 95% ethanol. 50μl poly-HEMA solution was added to each well of a round-bottomed 96 well plate and allowed to evaporate. This coating process was repeated once more and plates were allowed to dry completely before addition of cells. BT474 and EFM192A cells were seeded at 5×10^3^ cells/200μl/well, while HCC1954 cells were seeded at 3×10^3^ cells/200μl/well. Once seeded, 3D cell plates were centrifuged at 146*g* for 5 minutes. 50μl of medium was removed and replaced with fresh medium every second day.

### Scanning electron microscopy (SEM) imaging

SEM images were taken 2D and 3D cells. For 2D SEM images, 3×10^3^ cells in 200μl medium was placed on a sterile, poly-d-lysine (Sigma-Aldrich) coated coverslip and were allowed to attach overnight. The next day, medium was removed and cells were washed with 200μl PBS (Sigma-Aldrich) prior to processing for SEM. For 3D cultures, cells were seeded and grown for 7 days. Spheroids were collected in a 15ml tube (Corning) and allowed to settle to the bottom of the tube, washed with 200μl PBS and allowed to settle to the bottom of the tube once again. Cells were not centrifuged to prevent distortion of 3D structure of spheroids. PBS was removed from the tube, leaving approximately 100-200μl liquid in the tube with the cells. This small amount of liquid was pipetted, with the 3D cells, onto the poly-d-lysine coated coverslip and placed at 4°C for 30 minutes or until cells had attached to the coverslip.

For both 2D and 3D cells that were attached to coverslip, cells were fixed for 60 minutes with 200μl 3% glutaraldehyde (Agar Scientific) and washed 3 times with 200μl PBS for 10 minutes at 4°C. Cells were then dehydrated in serial increasing ethanol concentrations (10%, 30%, 50%, 70%, 95% and 100%) for 5 minutes each at room temperature. Dehydration was completed with a 5 minute incubation with 50% hexamethyldisilazane (Sigma-Aldrich) in ethanol at room temperature and a final 5 minute incubation with 100% hexamethyldisilazane at room temperature. Cells were gold sputter-coated using an EMScope SC500 Sputter Coater at 25mA for 3 minutes (approximately 15nm coating) and examined at various magnifications in a Tescan Mira XMU Field Emission Scanning Electron Microscope at 5Kv using a secondary electron detector.

### Viability of 2D and 3D cells measured through ATP

BT474 and EFM192A cells were seeded at 5×10^3^ cells/200μl/well and HCC1954 cells were seeded at 3×10^3^ cells/200μl/well, for both 2D and 3D cultured cells. 6 days after seeding, cell viability was determined by measuring cellular ATP levels using CellTitre-Glo 3D (Promega). This timing of 6 days was chosen so that the timing of these assays directly match the timing of the cells in culture (in 2D and 3D format) for the drug toxicity assays (described below). Luminescence was read using a Luminoskan plate reader (Thermo Scientific).

### Drug toxicity assays

2D and 3D cells were seeded as per cell viability assay. 24 hours after seeding, 2D and 3D cells were treated with the same fixed concentrations of neratinib and docetaxel. Approximate IC_50_ concentrations of drugs were used as established previously in our lab by the acid phosphatase method. Concentrations of neratinib used were: 2.5nM – BT474 cells, 49nM – HCC1954 cells and 6.8nM – EFM192A cells. Concentrations of docetaxel used were: 2.8nM – BT474 cells, 0.6nM – HCC1954 cells and 2.6nM – EFM192A cells. 5 days after treatment, cell viability was determined by measuring cellular ATP levels using CellTitre-Glo 3D (Promega).

### Immunoblotting

30μg total cellular sample protein was used for Akt, pAkt, Erk, pErk EGFR, pEGFR, HER2, pHER2, HER3, Caspase 3, Caspase 7, Caspase 9, PARP, CYP3A4, ALDH, β-catenin and E-cadherin, while 50μg total cellular sample protein was used for HER4, PGP and BCRP immunoblots. Proteins were resolved on 7.5 – 10% gels and were transferred to PVDF membrane and blocked using BSA. Blots were incubated with primary antibodies at 4°C overnight: HER2 (Calbiochem; 1:500), phosphorylated HER2 (pHER2) (Cell Signalling; 1:1000), EGFR (Cell Signalling; 1:1000), phosphorylated EGFR (pEGFR) (Cell Signalling; 1:1000), HER3 (Cell Signalling; 1:1000), HER4 (Cell Signalling; 1:1000), PGP (Santa Cruz; 1:200), BCRP (Santa Cruz; 1:200), E-Cadherin (Cell Signalling; 1:1000), ALDH (BD; 1:200), β-catenin (Cell Signalling; 1:1000), Caspase 3 (Cell Signalling; 1:1000), Caspase 7 (Cell Signalling; 1:1000), Caspase 9 (Cell Signalling; 1:1000), PARP (Cell Signalling; 1:1000), CYP3A4 (Santa Cruz; 1:200) and β-actin (Sigma-Aldrich; 1:1000). Blots were washed in PBS tween (0.1%) and were incubated for one hour at room temperature with the relevant horseradish peroxidase-conjugated secondary antibodies: anti-rabbit (Cell Signalling; 1:1000), anti-mouse (Cell Signalling; Cat. 1:1000) and anti-rat (Santa Cruz; 1:1000). Protein bands were visualised by chemiluminescence (ThermoFisher). Semi-quantitative densitometry analysis was performed on the bands of protein detected by immunoblot using ImageJ software and normalised to β-actin which served as a loading control.

### CYP3A4 activity in 2D and 3D cells

For cells, BT474 and EFM192A cells were seeded at 5×10^3^ cells/200μl/well and HCC1954 cells were seeded at 3×10^3^ cells/200μl/well, for both 2D and 3D cell cultures. 6 days after seeding, CYP3A4 activity was determined using P450-Glo CYP3A4 assay kit (Promega).

## SUPPLEMENTARY FIGURES


