# Supplementary material for: The relevance of using 3D cell cultures, in addition to 2D monolayer cultures, when evaluating breast cancer drug sensitivity and resistance
Source: Oncotarget. 2016 Jun 10;7(29):45745–56. doi: 10.18632/oncotarget.9935 (PMC5216757; doi:10.18632/oncotarget.9935)
Supplement: Supplementary file 1 [file oncotarget-07-45745-s001.pdf]

The relevance of using 3D cell cultures, in addition to 2D monolayer cultures, when evaluating breast cancer drug sensitivity and resistance

SUPPLEMENTARY FIGURES

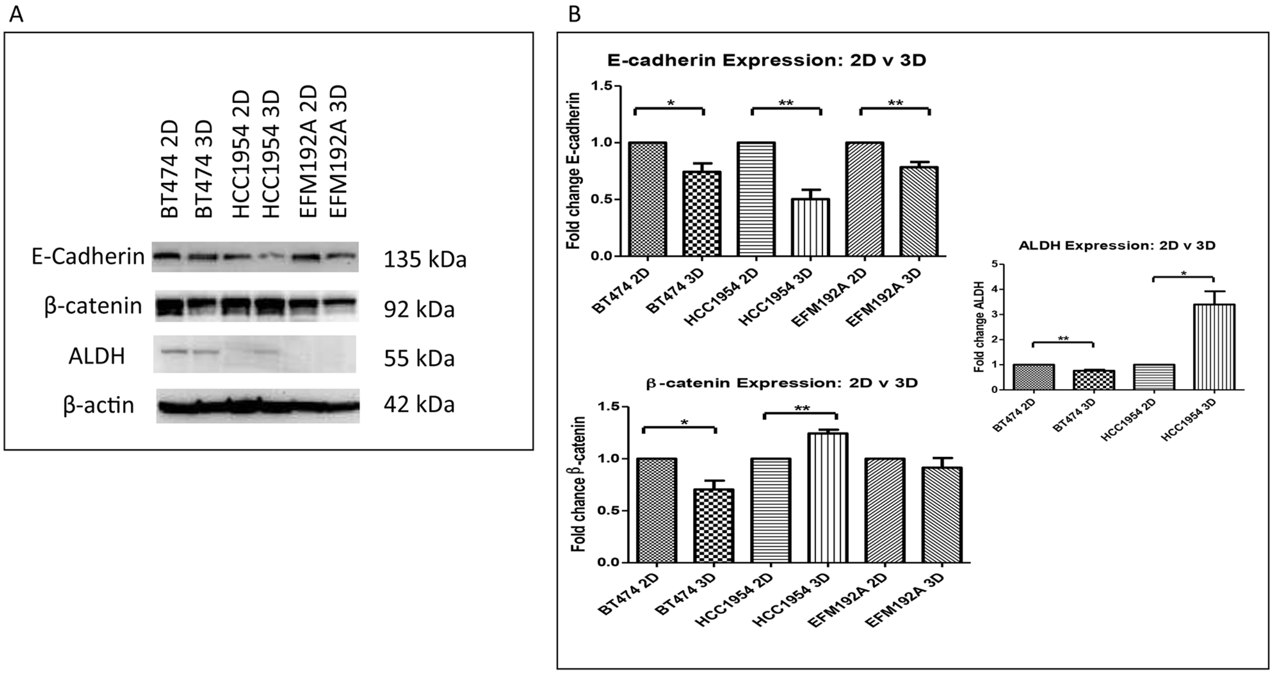

Supplementary Figure S1: A. Immunoblots of E-Cadherin, β-catenin and ALDH grown in 3D compared to 2D cells. **B.** Densitometry of respective immunoblots. Graphs represent triplicate biological repeats and are displayed as mean ± SEM, where *$p < 0.05$; **$p < 0.01$; **$p < 0.001$. Here we assessed the expression of E-cadherin, a cell-cell adhesion molecule. Unexpected, E-cadherin expression levels were decreased in 3D compared to 2D cells i.e. E-cadherin decreased to 0.74±0.07 (p=0.026), 0.5±0.1 (p=0.004) and 0.78±0.05 (p=0.01) in BT474 3D, HCC1954 3D and EFM192A 3D cells in comparison to their respective 2D counterparts. We also analysed the expression of two EMT-associated markers, ALDH and β-catenin. While we observed a 3.4±0.5 fold (p=0.01) increase in ALDH expression in HCC1954 3D compared to 2D cells, ALDH expression in BT474 3D cells decreased to 0.76±0.05 (p=0.006). ALDH was not detected in EFM192A 2D or 3D cells. We found that β-catenin decreased to 0.7±0.08 (p=0.025) and 0.9±0.03 (p=0.407) in BT474 and EFM192A 3D cells compared to cells grown in 2D, while HCC1954 3D cells had 1.24±0.04 fold (p=0.002) increase in β-catenin compared to 2D cells. Thus, there is no specific trend observed here regarding EMT and 3D cell culture.

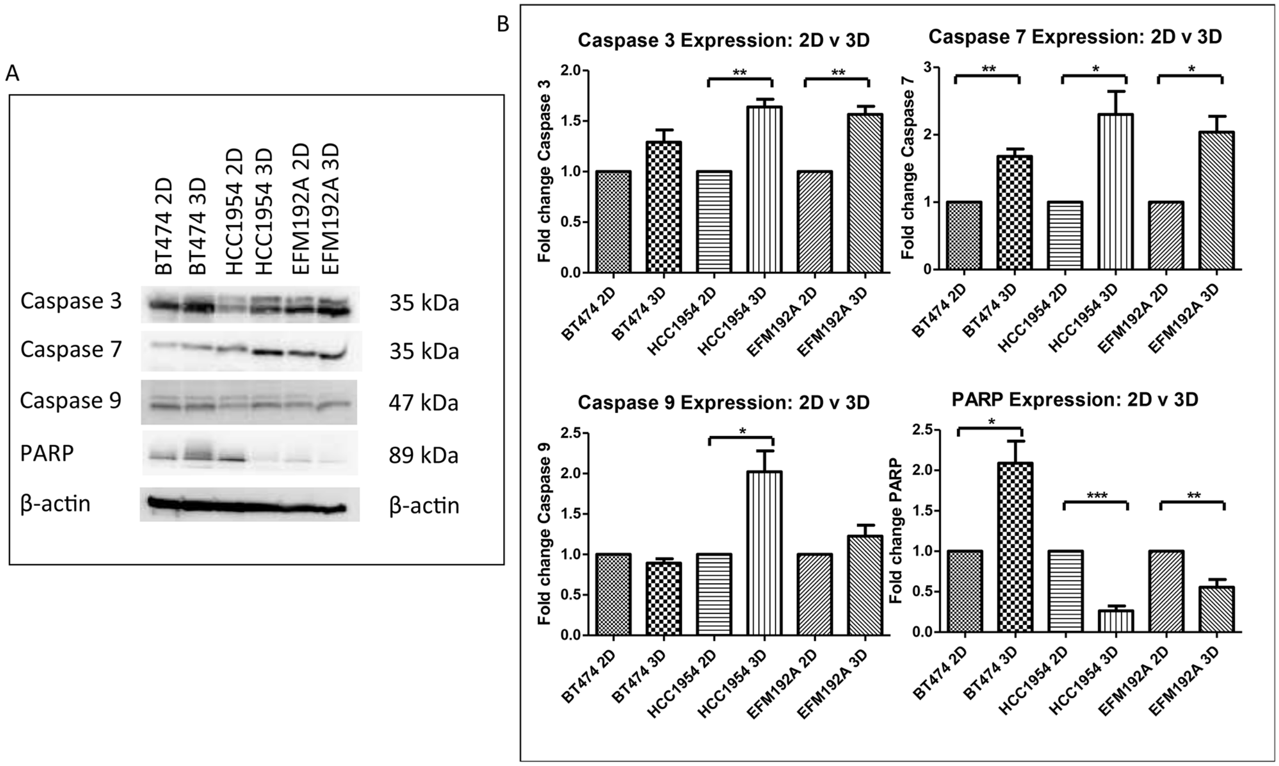

Supplementary Figure S2: A. Immunoblots for Caspase 3, Caspase 7, Caspase 9 and PARP in cells grown in 3D compared to 2D cells. **B.** Densitometry of respective immunoblots. Graphs represent triplicate biological repeats and are displayed as mean ± SEM, where **p* < 0.05; ***p* < 0.01; ***p* < 0.001. We investigated a number of apoptotic markers to determine the influence of growing cells in 3D on expression of these proteins. Caspase 8 was found to be increased by 1.3±0.1 fold (p=0.076), 1.64±0.08 fold (p=0.001) and 1.57±0.08 fold (p=0.002), in BT474 3D, HCC1954 3D and EFM192A 3D cells compared to their respective 2D counterparts. Caspase 7 was increased by 1.68±0.1 fold (p=0.004), 2.3±0.3 fold (p=0.019) and 2.04±0.2 fold (p=0.01) in BT474 3D, HCC1954 3D and EFM192A 3D cells compared to 2D counterparts. Caspase 9 was increased by 2.02±0.3 fold (0.017) in HCC1954 3D compared to 2D cells, but there was no significant difference in Capase 9 levels in BT474 3D and 2D cells (decreased to 0.89±0.5 (p=0.166) or EFM192A 3D and 2D cells (increased by 1.2±0.13 fold (p=0.166)). PARP was increased to 2.1±0.3 fold (p=0.017) in BT474 3D cells compared to BT474 2D, while it was decreased to 0.26±0.06 ($p=2.5\times10^{-4}$) and 0.6±0.1 (p=0.01) in HCC1954 3D and EFM192A 3D cells compared to their 2D counterparts.
